# Physico-chemical characterization of *Anopheles gambiae s.l.* breeding sites and kdr mutations in urban areas of Cotonou and Natitingou, Benin

**DOI:** 10.1186/s12879-024-09440-8

**Published:** 2024-05-30

**Authors:** Innocent Djègbè, Donald Hessou-Djossou, Massioudou Koto Yerima Gounou Boukari, Odilon Nonfodji, Geneviève Tchigossou, Rousseau Djouaka, Sylvie Cornelie, Martin Akogbeto, Luc Djogbenou, Fabrice Chandre

**Affiliations:** 1 Département des Sciences de la Vie et de la Terre, Ecole Normale Supérieure de Natitingou, Natitingou, Bénin; 2 Plateforme Agriculture Environnement Santé, Institut International d’Agriculture Tropicale (IITA-Bénin), Cotonou, Bénin; 3 Laboratoire de Chimie de l’Eau et de l’Environnement (LCEE), Ecole Normale Supérieure de Natitingou, UNSTIM, Natitingou, Bénin; 4 Evolution et Contrôle, UMR IRD 224-CNRS, Université de Montpellier2. MIVEGEC. Maladies Infectieuses et Vecteurs : Ecologie, Génétique, Montpellier cedex 5, 5290 France; 5 Centre de Recherche Entomologique de Cotonou (CREC), Ministère de la Santé, Cotonou, Bénin; 6 Institut Régional de Santé Publique (IRSP), Université d’Abomey-Calavi (UAC), Ouidah, Bénin

**Keywords:** Malaria, *Anopheles gambiae*, Breeding sites, Physico-chemical properties, Kdr, Benin

## Abstract

**Background:**

This study aimed to investigate the relationship between the physicochemical characteristics of *An. gambiae s.s. and An. coluzzii* breeding sites, the susceptibility profiles to commonly used insecticides in public health, and the underlying insecticide resistance mechanisms.

**Methods:**

Anopheles breeding sites surveys were conducted in Cotonou and Natitingou in September 2020, January and August 2021. Physicochemical properties and bacterial loads were determined in individual breeding sites. The WHO susceptibility assays were carried out using the female of the emerging adult mosquitoes. Anopheles species were identified through PCR techniques. *Kdr L1014F/S*, *N1575Y* and *G119S* mutations were investigated using TaqMan genotyping assays.

**Results:**

Molecular analysis showed that all mosquitoes analyzed in Cotonou were *Anopheles coluzzii*, while those of Natitingou were *Anopheles gambiae s.s.* Fecal coliforms were identified as playing a role in this distribution through their significant influence on the presence of *An. coluzzii* larvae. WHO susceptibility assay indicated a high level of resistance to deltamethrin in the two cities. The resistance levels to deltamethrin were higher in Cotonou (X^2^ = 31.689; DF = 1; *P* < 0.0001). There was a suspected resistance to bendiocarb in Cotonou, whereas the mosquito population in Natitingou was resistant. The *kdr L1014F* mutation was highly observed in both mosquito populations (frequence: 86–91%), while the Ace-1 mutation was found in a small proportion of mosquitoes. In Cotonou, salinity was the only recorded physicochemical parameter that significantly correlated with the resistance of Anopheles mosquitoes to deltamethrin (*P* < 0.05). In Natitingou, significant correlations were observed between the allelic frequencies of the *kdr L1014F* mutation and pH, conductivity, and TDS.

**Conclusion:**

These results indicate a high level of pyrethroid resistance in the anopheles populations of both Cotonou and Natitingou. Moreover, this study report the involvement of abiotic factors influencing Anopheles susceptibility profile.

## Introduction

Malaria stands as the most significant parasitic disease in public health worldwide [1]. From 2020 to 2021, the incidence of malaria cases rose from 245 million to 247 million, 93% of recorded cases occurred in Africa [1]. This alarming situation underscores the urgent need to intensify prevention and control efforts across the continent. Malaria control primarily involves managing three interconnected entities: humans (the secondary host), Anopheles mosquitoes (the primary host), and the aquatic stages of the vector (eggs, larvae, and pupae) confined to specific habitats [2].

Historically, Sub-Saharan African (SSA) countries have focused their preventive approaches on targeting adult mosquitoes to reduce malaria incidence. These strategies have encompassed the use of insecticide-treated nets (ITNs), primarily reliant on pyrethroids, and indoor residual spraying (IRS) [3, 4]. If initially, these efforts greatly contributed to reduce the malaria burden, the impact of these approaches on disease burden has been limited, particularly in urban areas experiencing an escalating malaria problem and a significant number of annual clinical episodes [5]. Various factors contribute to this, including the rapid spread of vector resistance to insecticides due to urban agricultural expansion, uncontrolled urbanization process, and population growth [6, 7]. Consequently, a thorough evaluation of malaria transmission risks across diverse ecological settings is imperative. Additionally, there is a necessity to investigate alternative methods for malaria vector control, with a particular focus on targeting the immature stages of mosquitoes, either as standalone interventions or as part of integrated vector management strategies. These efforts will aim to reduce malaria transmission intensity in urban areas [7, 8].

Several studies have demonstrated that the establishment of effective larval control methods must consider the dynamics associated with various environmental elements, such as climate and physicochemical properties, influencing larval development and distribution [9, 10]. The impact of environmental factors on mosquito oviposition, larval density, and development has been reported in malaria endemic areas of Africa. Recently, a previous study conducted by our research team revealed a significant positive correlation between larval density and temperature, dissolved oxygen, and salinity in the coastal zone of Benin [11]. Additionally, Ukubuiwe et al. [12] found that mosquito larvae can adapt to higher ambient pH levels than other aquatic organisms. In Western Kenya, a study showed a positive correlation between anopheline abundance and nitrate levels in water, while negative but significant correlations were observed with iron levels in water and biofilm cover on the water surface [13]. Furthermore, microorganisms have been highlighted as influential factors affecting mosquito larval distribution, serving as a food source but also potentially impacting mosquito physiology. They may also play a role in attracting or repelling ovipositing female mosquitoes.

In Benin, similar to other Sub-Saharan African countries, a high prevalence of malaria vectors resistance to commonly used insecticide classes, particularly pyrethroids, has been reported in numerous cities [14–18]. The primary mechanisms underlying this resistance involve an increase in the activity of detoxification enzymes (oxidases, esterases, and glutathione-S-transferases) and the presence of *kdr L1014F* and *G119S Ace-1* target site mutations, frequently found in *An. gambiae* (s.l.) populations [19]. Furthermore, an additional mutation, *N1575Y*, has been documented to a lesser extent in the country and appears to have a synergistic effect on pyrethroid resistance [20, 21]. Moreover, it is essential to explore the distribution and abundance of Anopheles species in relation to climate features, as well as the possible association between abiotic factors of breeding sites and insecticide resistance mechanisms in mosquito. For instance, few studies investigated the physicochemical properties of Anopheles breeding sites, their resistance status and associated mechanisms in Benin. Therefore, this study aims to assess the correlation between physicochemical characteristics of *An. gambiae s.l.* breeding sites, and susceptibility to common insecticides in Cotonou and Natitingou, two major urban cities of Benin.

## Materials and methods

### Study areas

**Fig. 1:**
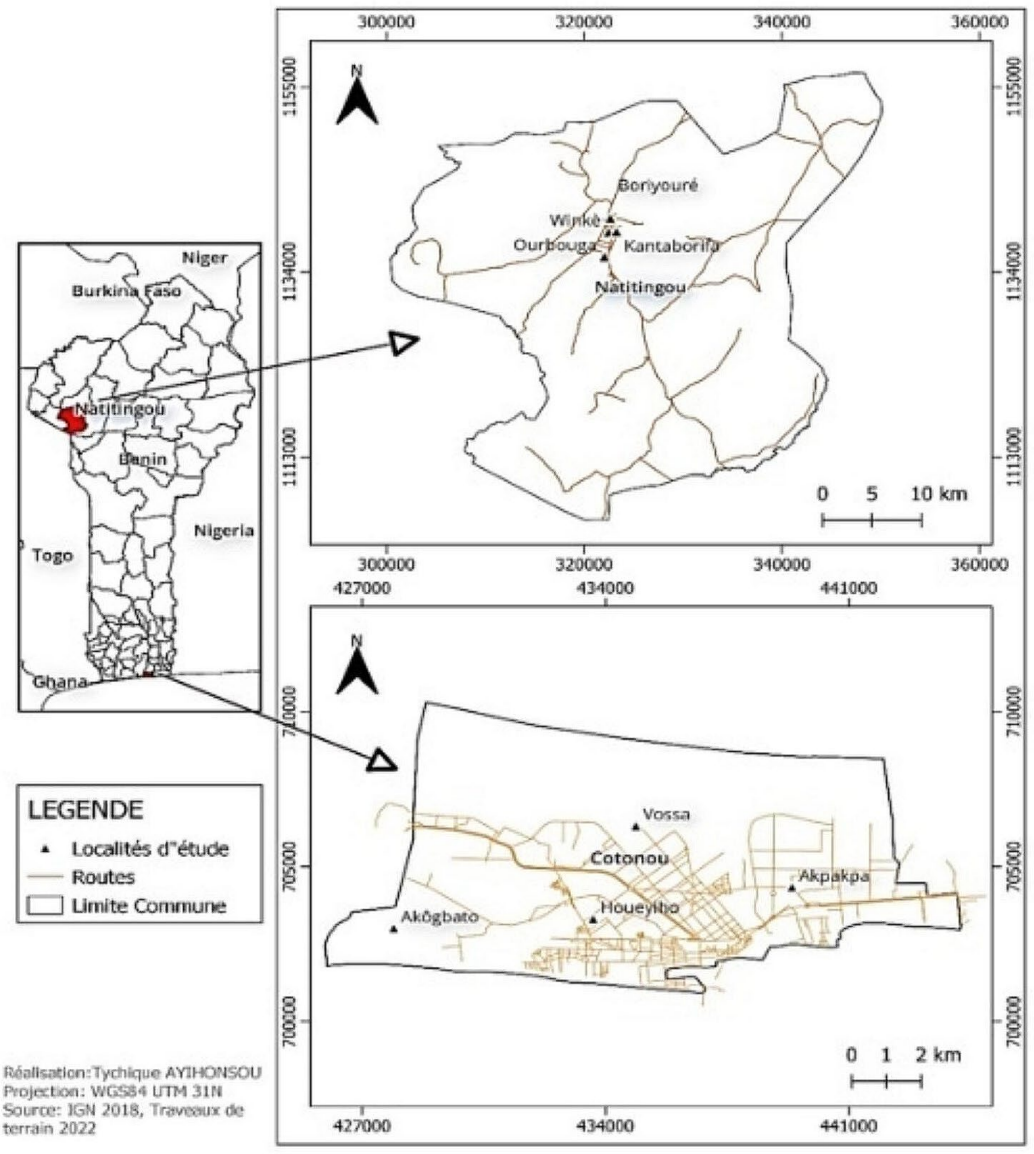
Map of study areas

The study was conducted in the two major cities of southern and northern of Benin namely Cotonou and Natitingou (Fig. 1). The two sites were chosen because of the relatively divergent human activities in them. Indeed, in Cotonou, the predominant human activities include bustling markets, industrial zones, and urban settlements. The city’s proximity to the coast also influences activities such as fishing and port-related operations. Additionally, Cotonou experiences high levels of traffic congestion and pollution due to its dense population and industrial activities [22]. Whereas in Natitingou, agricultural activities play a significant role in the local economy, with farming communities engaged in crop cultivation and livestock rearing. The city’s surroundings consist of rural landscapes, with fewer urban developments and infrastructures compared to Cotonou [23].

From geographical standpoint, Cotonou is located within the coastal strip that stretches between Lake Nokoué and the Atlantic Ocean, offering unique characteristics. The city hosts a tropical savanna climate with two distinct rainy seasons annually. The first rainy season spans from March to June, followed by a second rainy season from September to November. Precipitation varies between 900 and 1200 mm, while the average temperature fluctuates between 25 °C and 32 °C, with higher humidity levels prevalent throughout the year. The landscape of Cotonou is not very rugged and includes swamps and lowlands. The city of Cotonou covers an area of 79 km^2^ and has a population of around 1,300,000 inhabitants [24]. The districts selected for larval collection were Houeyiho, Akpakpa, Vossa and Akogbato.

Natitingou, located in northwestern Benin, has a rugged terrain and is situated at an altitude of 500 m above sea level. Covering an area of 3045 km^2^ with a population of approximately 103,843 inhabitants [24], the city experiences a Sudano-Guinean climate characterized by a single rainy season annually from May to October. This period brings abundant rainfall to the region, with some years recording up to 1400 mm of precipitation. The high annual rainfall supports agricultural activities, contributes to water resources, and shapes the natural landscape of the city. The larval collection was carried out in the following districts: Winkè, Ourbouga, Kantaborifa and Boriyouré.

### Mosquito larval sampling

Anopheles larvae collection was carried out in the selected study sites in Cotonou and Natitingou between 10 a.m. to 5 p.m. during one week in September 2020, January and August 2021. At each site, collections took place in both urban and non-urban districts, with two districts of each type included.

The sampling were performed using standard dipping method with 350 ml dipper. Presence or absence of mosquito larvae was determined after 10 to 15 dips. The larvae were identified on the basis of their spatial projections on the surface of the waters (horizontally inclined). If larvae were present, they were transferred to other plastic containers which were then loosely capped to allow aeration. The number of larvae and pupae were recorded, and the larval density was estimated as the ratio of the number of larvae collected per dip [25, 26].

### Physicochemical and bacterial properties of larval habitats

During the larval collection, physicochemical parameters of each positive breeding site were measured. This included pH using Hanna HI 991,001 pH meter; turbidity using a Hanna HI 93,703 Turbidity Meter; dissolved oxygen (DO) using WTW OXI 3205 oximeter; conductivity, total dissolved solids, salinity and temperature using VWR CO300 multi-parameter meter. After the measurement, at least 200 mL of water was taken from each breeding site and placed into a sterile conical flask. Then, the flask was placed into an icebox and transported to the laboratory to analyze the bacterial pollution of larval habitats.

The level of microbiological pollution of the breeding sites was evaluated by isolation and identification of total coliforms and *Escherichia coli* at 37 °C and faecal coliforms at 44 °C. The water was filtered through a 0.45-µm nitrocellulose membrane and the microorganisms cultured on Chromogenic Coliform Agar (CCA) according to the protocol described by Nonfodji et al. [27].

### Mosquito rearing and insecticide susceptibility testing

All the collected larvae and pupae from different sites were transported to the insectary of the “Ecole Normale Supérieure de Natitingou” for rearing. They were fed with TetraMin fish food and reared under standard insectary conditions (temperature of 27 °C ± 2 °C and relative humidity of 75% ± 5%). Adult mosquitoes that emerged were provided with 10% honey solution for growing before selecting and subjecting the females for the bioassay.

Protocols and standard insecticide-treated papers supplied by WHO were used to evaluate susceptibility profile of Anopheles populations from different sites to various insecticides classes [28] namely, pyrethroids type II, deltamethrin (0.05%), the organophosphate, pirimiphos methyl (0.25%) and the carbamate, bendiocarb (0.1%). For each dose of insecticide, approximately 20 non-blood-fed female mosquitoes were introduced into each tube lined with insecticide-impregnated paper. For each bioassay, six test tubes were used: two control (impregnated with acetone) and four containing impregnated papers. After 60 min of exposure, the mosquitoes were transferred to observation tubes containing untreated paper, with free access to 10% honey solution.

At the end of the tests, alive and dead specimens were used for molecular identification of species and screening of resistance mechanisms.

### Identification of anopheles mosquitoes

Anopheles mosquitoes were identified using a morphological key described by Gillies & Coetzee [29]. *An. gambiae s.l*. genomic DNA from both alive and dead mosquitoes was extracted using the DNA extraction protocol described by Livak [30]. Specific DNA sequences were amplified using SINE-PCR technique described by Santolamazza et al. [31]. to identify *An. gambiae s.l.* species.

### Molecular characterization of *kdr L1014F, L1014S, N1575Y* and *Ace‑1 G119S* resistance alleles

TaqMan assays with two labelled fluorochromes probes HEX/VIC and FAM were used to assess the presence of *L1014S*, and *L1014F kdr* mutations [32], *N1575Y* mutation [33] on the voltage gated sodium channel gene and *G119S* mutation on the *Ace-1* gene [14, 34]. All assays used these two probes; the first (HEX/VIC) was specific for the wildtype allele and the second (FAM) was specific for the mutant allele. A substantial increase in HEX/VIC fluorescence indicates homozygous wildtype, substantial increase in FAM fluorescence indicates homozygous mutant and, usually intermediate, increase in both signals indicates heterozygote. Amplifications were performed in an Agilent MX3000 real-time qPCR machine (Agilent Technologies, Santa Clara, CA, USA). The genotype was determined from the fluorescence profiles and bi-directional scatter plots generated in the MX3005P software.

### Data analysis

World Health Organization criteria [28] were used to determine phenotypic resistance status of mosquito population as follows: Mortality rate > 98% (susceptible mosquito population); Mortality rates ranged between 90 and 98% (suspected resistance in the mosquito population); Mortality rates < 90% (mosquito population resistance to the insecticide). Genotypes distributions were recorded in an Excel datasheet and analysis performed using SPSS 25.0. Allelic frequencies were calculated using the following formula ƒ(R) = (2n.RR + n.RS)/2n, where n is the number of mosquitoes of a given genotype, RR represents the homozygote resistance allele, RS represents the heterozygote resistance allele, SS the susceptible allele, and n is the total number of mosquitoes tested. Chi-square test (X^2^) was used to compare insecticide resistance profiles in different breeding sites and between study cities. The relationship between water properties, distribution of *An. gambiae* species, insecticide resistance profiles and *kdr* alleles, was determined using Pearson bivariate correlation analysis and Welch’s test. All levels of statistical significance were determined at *P* < 0.05.

## Results

### Characteristics of anopheles’ larval habitats

General characteristics of mosquito larval habitats in the two cities are presented in Table 1. A total of 156 breeding sites including 88 (56.4%) breeding sites in Cotonou and 68 (43.6%) in Natitingou were sampled in the two collections periods. During the rainy season, a total of 139 (89.1%) larval habitats were recorded, while only 17 (10.9%) were encountered in the dry season. Furthermore, a higher and statistically significant proportion of larval habitats were recorded in urban areas compared to non-urban environment areas in both study cities (Cotonou: χ^2^ = 4.301, df = 1, *P* = 0.0381; Natitingou: χ^2^ = 18.526, df = 1, *P* < 0.0001). The typology of the encountered breeding sites encompasses gutters, tires, vegetable farms, puddles, tire tracks, swamps, pits, water containers, hoof imprints, cans, ponds, wells, hollow bricks, construction sites, etc. This classification enables the categorization of these sites into permanent or temporary habitats and natural or artificial.

The majority of breeding sites in the two cities were temporary regardless the season of collection with a significant difference during the rainy season: 87.3% (χ^2^ = 24.075, df = 1, *P* < 0.0001) and 85.7% (χ^2^ = 20.923, df = 1, *P* < 0.0001) at Cotonou and Natitingou, respectively. Moreover, the comparison of these characteristics between urban and non-urban areas within the two study cities revealed no statistically significant differences (*P* > 0.05).

### Properties of *An. gambiae s.l.* breeding habitats

The properties of the breeding sites are presented in Tables 2 and 3. A total of ten parameters of water including 07 physico-chemical and 03 bacterial were measured. The result showed a significantly higher temperature, pH, conductivity, and TDS in Cotonou than those of Natitingou (*P* < 0.05). On the other hand, turbidity and dissolved oxygen were significantly higher at Natitingou. Moreover, all bacterial loads were higher in larval habitats found in Natitingou compared to those in Cotonou.

Within the same city, differences were observed between the characteristics of larval habitats depending on whether they were found in urban or non-urban areas. At Cotonou, temperature, pH and bacterial loads were significant higher in urban breedings sites in comparison to non-urban breeding sites (*P* < 0.05). At Natitingou, only the faecal coliform load of non-urban breedings sites was statistically different from those of urban breedings sites (*P* = 0.0062).

**Table 1:** Characteristics of breeding sites sampled in Cotonou and Natitingou

Seasons	Cities		Total of breeedig sites	Breeding sites with anopheles & culicidae *n* (%)	Nature		Origin	
					Temporary *n* (%)	Permanent *n* (%)	Natural *n* (%)	Artificial *n* (%)
Rainy season	Cotonou	Non-urban areas	28	8 (28.6%)	26 (92.8%)	2 (7.2%)	21 (75%)	7 (25%)
		Urban areas	48	9 (18.7%)	42 (87.5%)	6 (12.5%)	35 (72.9%)	13 (27.1%)
	Natitingou	Non-urban areas	13	9 (69.2%)	13 (100%)	0	4 (30.8%)	9 (69.2%)
		Urban areas	50	15 (30%)	41 (82%)	9 (18%)	31 (62%)	19 (38%)
Dry season	Cotonou	Non-urban areas	6	1 (16.7%)	3 (50%)	3 (50%)	3 (50%)	3 (50%)
		Urban areas	6	1 (16.7%)	6 (100%)	0	0	6 (100%)
	Natitingou	Non-urban areas	0	0	0	0	0	0
		Urban areas	5	2 (40%)	3 (60%)	2 (40%)	3 (60%)	2 (40%)
Total			156	45 (28.8%)	134 (85.9%)	22 (14.1%)	97 (62.2%)	59 (37.8%)

**Table 2:** Physico-chemical and bacterial characteristics of breeding sites in Cotonou and Natitingou

	Temperature (^o^C)	pH	Turbidity (NTU)	Conductivity (µS/cm)	Dissolved oxygen (mg/L)	Salinity (g/L)	TDS (mg/L)	Total coliforms (UFC/100mL)	Fecal coliforms (UFC/100mL)	E. coli (UFC/100mL)
Cotonou	32.01 ± 3.83	8.13 ± 0.91	96.01 ± 147.71	341.82 ± 235.0	2.58 ± 1.11	0.10 ± 0.08	215.28 ± 151.53	168,250 ± 206867.20	160938.71 ± 27701.62	18,450 ± 55874.37
Natitingou	28.07 ± 2.17	7.48 ± 0.47	174.9 ± 196.19	254.04 ± 147.2	3.85 ± 1.60	0.12 ± 0.07	152.0 ± 92.47	228,041 ± 188438.02	12,425 ± 22925.59	22754.84 ± 25609.21
*t-test*	-7.590	-5.361	2.866	-2.697	5.847	1.634	-3.033	1.860	1.613	0.634
*P value*	< 0.0001	< 0.0001	0.0047	0.0078	< 0.0001	0.1043	0.0028	0.0647	0.1087	0.5268

**Table 3:** Physico-chemical and bacterial characteristics of breeding sites from urban and non-urban areas in Cotonou and Natitingou

Parameters	Cotonou			Natitingou		
	Non-urban breeding sites	Urban breeding sites	*P* value	Non-urban breeding sites	Urban breeding sites	*P* value
Temperature (°C)	29.8 ± 3.74	33.2 ± 3.41	< 0.0001	28.75 ± 2.69	27.79 ± 1.92	0.1420
pH	7.77 ± 0.85	8.33 ± 0.90	0.0047	7.62 ± 0.47	7.41 ± 0.46	0.1467
Turbidity (NTU)	78.64 ± 102.83	105.36 ± 168.17	0.4074	247.66 ± 192.85	145.1 ± 193.96	0.0922
Conductivity (µS/cm)	384.4 ± 143.59	317.98 ± 273.17	0.1948	287.42 ± 196.53	240.4 ± 124.77	0.2853
Dissolved oxygen (mg/L)	2.62 ± 1.16	2.56 ± 1.10	0.8078	3.68 ± 1.37	3.93 ± 1.71	0.6265
Salinity (g/L)	0.11 ± 0.08	0.10 ± 0.08	0.5695	0.13 ± 0.10	0.11 ± 0.05	0.3051
TDS (mg/L)	246.79 ± 93.31	198.32 ± 174.46	0.1401	179.97 ± 116.09	140.58 ± 81.30	0.1570
Total coliforms (UFC/100mL)	203285.71 ± 181674.02	149384.61 ± 220314.15	< 0.0001	308333.33 ± 170800.32	155195.45 ± 188998.44	0.0062
Fecal coliforms (UFC/100mL)	23807.14 ± 34159.86	6296.15± 10075.13	< 0.0001	23716.67 ± 29480.67	14165.90± 27155.56	0.2678
*E. coli* (UFC/100mL)	44714.28 ± 87417.15	4307.69 ± 17384.52	< 0.0001	28133.33 ± 24317.74	20,554 ± 26346.83	0.3494

### Species composition

A total of 618 specimens of *An. gambiae (s.l.)* from different breeding sites surveyed in Cotonou and Natitingou were analyzed by PCR. In Cotonou, all the 243 mosquitoes analyzed were identified as *Anopheles coluzzii* while all the 375 mosquitoes from Natitingou were identified as *Anopheles gambiae s.s.*

### Physico-chemical characteristics and species distribution

**Fig. 2:**
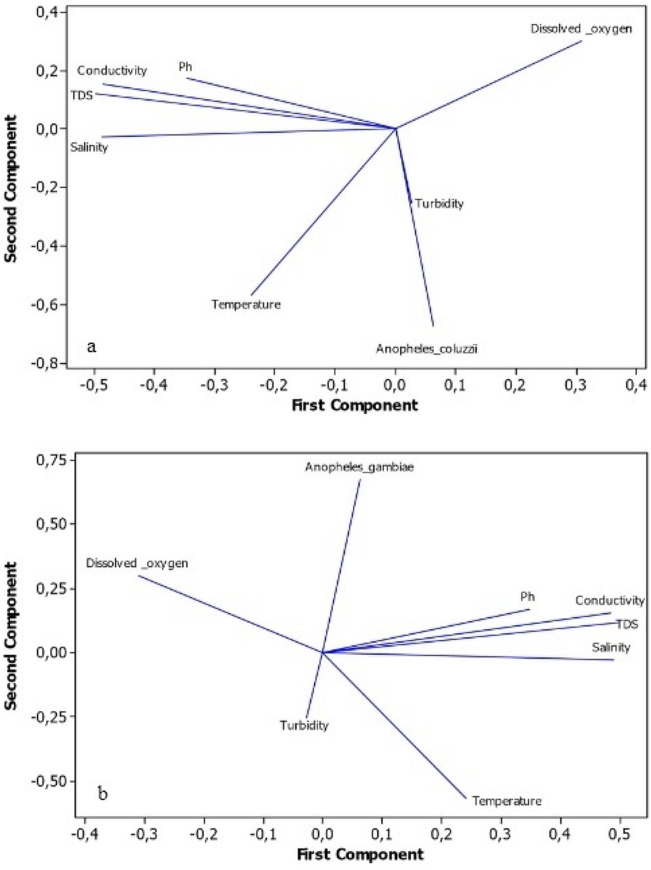
Principal component analysis (PCA) of physicochemical variables associated with proportions of (**a**) *Anopheles coluzzii* and (**b**) *Anopheles gambiae*

Physico-chemical data recorded in the habitats with the species of the *An. gambiae* complex identified revealed that parameters such as the temperature (*P* = 0.0019; *r* = 0.63) and the turbidity (*P* = 0.027; *r* = 0.56) had a significant impact on the presence of *An. coluzzii* (Fig. 2). On the other hand, the presence of *An. gambiae s.s.* in larval habitats was negatively and significantly impacted by turbidity (*P* = 0.001; t = -0.69).

### Bacterial loads and species distribution

**Fig. 3:**
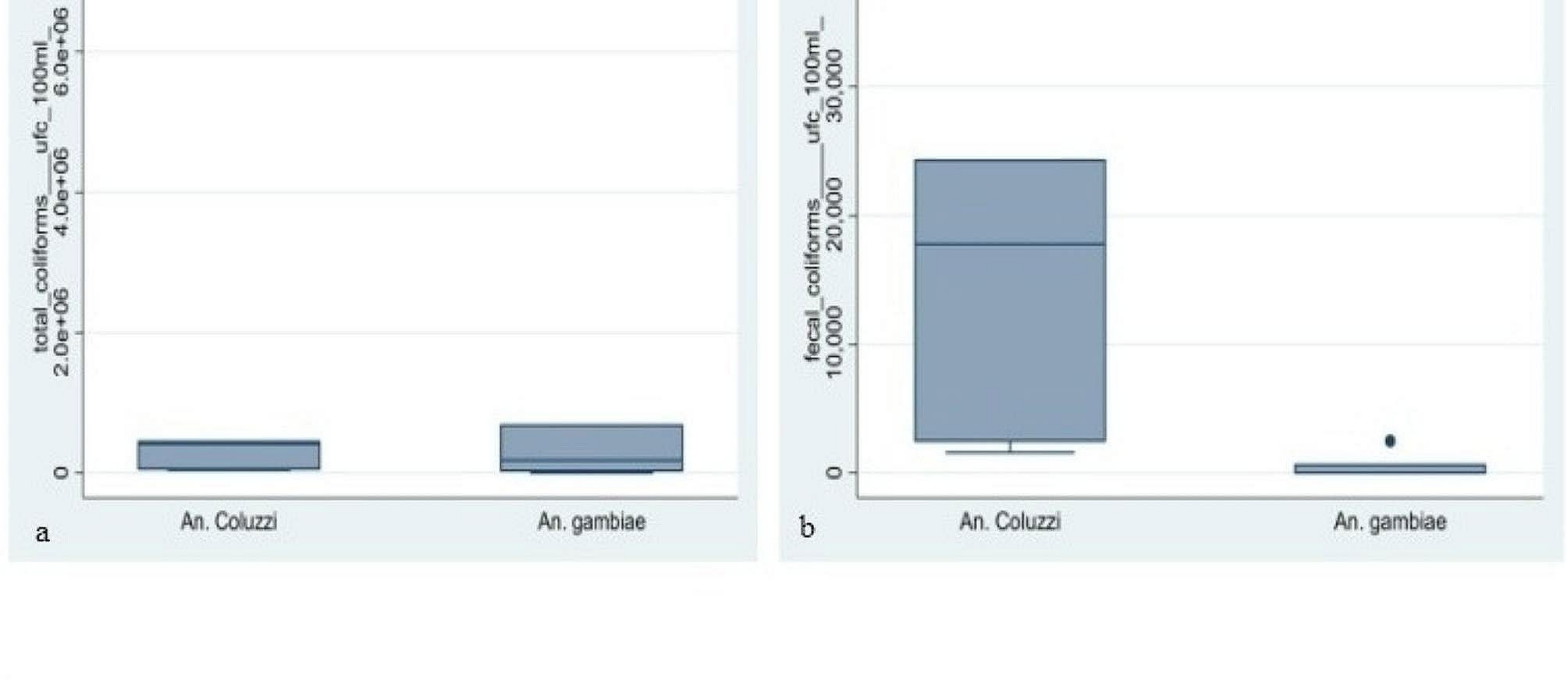
Logarithmic load of (**a**) total coliforms and (**b**) fecal coliforms in relation to *Anopheles gambiae s.l.* species in larval habitats

Figure 3 shows that the load of total coliforms in larval habitats was more significant in those harboring *An. gambiae s.s.* larvae compared to those of *An. coluzzii*. However, the Welch’s t-test reveals that the load of Total coliforms and the presence of *An. gambiae s.s.* were not linked (*P* = 0.1319; t = -1.5090). Conversely, the analysis of the graph indicates that the load of fecal coliforms was more substantial in the larval habitats of *An. coluzzii*, and the Welch’s t-test demonstrates a significant impact of these coliforms on the presence of *An. coluzzii* (*P* = 0.0000; t = 15.2367).

According to the results of the statistical analysis, the load of *E. coli* in different larval habitats didn’t have an impact on the presence and distribution of *An. gambiae s.s.* in the breeding sites (*P* = 0.2331; t = -1.19).

### Insecticide resistance status

**Fig. 4:**
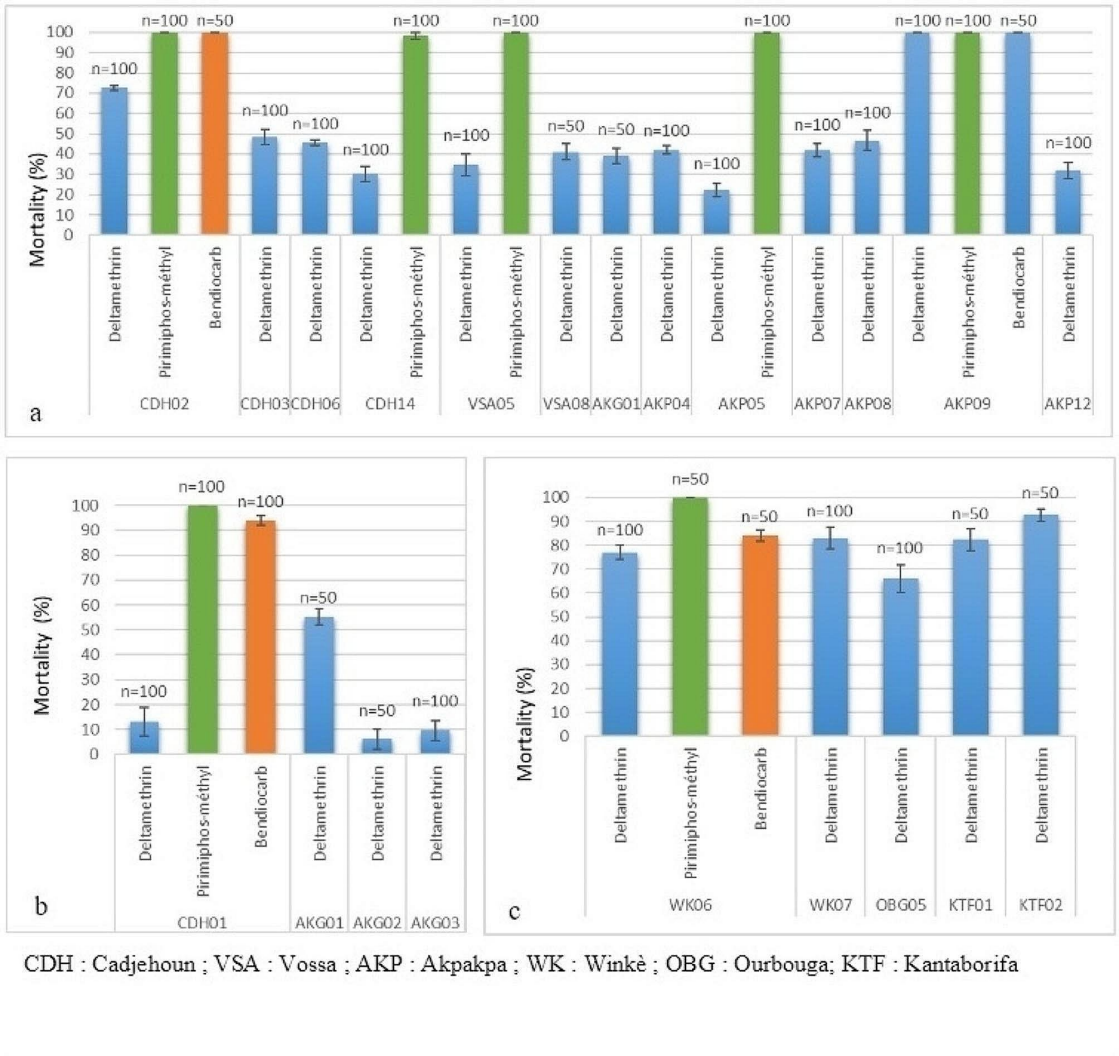
Insecticide resistance profiles of *Anopheles gambiae* population in Cotonou (**a** : rainy saison ; **b** : dry season) and Natitingou (c : rainy season)

The results of the WHO insecticide bioassay are shown in Fig. 4. It revealed high resistance levels of mosquitoes tested against deltamethrin in the two cities. Within the same city, the mosquitoes resistance levels varied from one district to another. In Cotonou, the mortality rates varied from 9.6 ± 4.04% to 72.52 ± 1.04% while in Natitingou it varied from 66 ± 5.81% to 92.62 ± 2.62%. Comparatively, the level of mosquitoes resistance to deltamethrin was higher in Cotonou than Natitingou (χ^2^ = 31.689; DF = 1; *P* < 0.0001).

A suspicion of resistance to bendiocarb (carbamate) was observed in Cotonou. The mortality rate was 93.83 ± 1.83%. In Natitingou the mortality rate to bendiocarb was 84.09 ± 2.27%, indicating a resistance of mosquito populations to bendiocarb in the city.

However, full susceptibility of mosquitoes to pirimiphos-methyl was observed in the two cities, with mortality rates over than 98%.

### Molecular characterization of insecticide resistance alleles

The *kdr L1014F* mutation was the main resistance mechanism observed in these populations of *An. gambiae* with a high frequency in mosquitoes from the city of Cotonou (0.91) and Natitingou (0.86) (Table 4). No significant difference was observed between the frequencies of the *kdr L1014F* mutation of these two cities (*P* > 0.05).

The *Kdr L1014S, N1575Y* mutations were not recorded in any specimens analyzed in either Cotonou or Natitingou. The *Ace-1* mutation was found but at very low frequencies in population of *An. coluzzii* from Cotonou (0.03) and *An. gambiae s.s.* from Natitingou (0.09) (Table 4).

**Table 4:** Allelic frequencies of mutations within different species of the *An. gambiae s.l.* complex across the study cities

Mutations	Cities	*Anopheles coluzzii*				*Anopheles gambiae s.s.*			
		Genotypes			Frequence	Genotypes			Frequence
		RR	RS	SS		RR	RS	SS	
***L1014F***	Cotonou	208	26	8	0.91	-	-	-	-
	Natitingou	-	-	-	-	272	102	0	0.86
***L1014S***	Cotonou	0	0	242	0	-	-	-	-
	Natitingou	-	-	-	-	0	0	374	0
***N1575Y***	Cotonou	0	0	242	0	-	-	-	-
	Natitingou	-	-	-	-	0	0	373	0
***Ace-1***	Cotonou	0	13	227	0.03	-	-	-	-
	Natitingou	-	-	-	-	0	68	284	0.09

### Physicochemical properties of larval habitats and distribution of pyrethroid resistance alleles in *An. gambiae* species

Data analysis revealed that salinity was the only recorded physicochemical parameter at which the Anopheles resistance to deltamethrin was significantly correlated (*r*= -0.645; *P* < 0.05).

In Natitingou, the bivariate correlation of the frequency of *kdr L1014F* alleles (homozygous and heterozygous) with the physicochemical properties of larval habitats shown a significant correlation between the allelic frequencies of *kdr L1014F* and pH (-0.848; *P* = 0.042), conductivity (0.853; *P* = 0.038) and TDS (0.854; *P* = 0.038) (Table 5).

**Table 5:** Correlation of physicochemical parameters with the frequency of the *kdr L1014F* mutation

Cities	Temperature	pH	Turbidity	Conductivity	Dissolved oxygen	Salinity	TDS
Cotonou	0.632 (*p* = 0.133)	0.223 (*p* = 0.175)	-0.199 (*p* = 0.205)	-0.79 (*p* = 0.09)	-0.483 (*p* = 0.188)	0.384 (*p* = 0.161)	-0.786 (*p* = 0.08)
Natitingou	-0.233 (*p* = 0.159)	-0.848 (*p* = 0.042)	0.136 (*p* = 0.240)	-0.853 (*p* = 0.038)	0.740 (*p* = 0.108)	-0.899 (*p* = 0.048)	-0.854 (*p* = 0.038)

## Discussion

The present study assessed intrinsinc breeding sites factors influencing the *An. gambiae s.l*. species distribution, and resistance to common insecticides used in the cities of Cotonou and Natitingou. *An. gambiae s.l.* larvae were found in various habitats including temporary water collections, permanent sites, artificial and polluted breeding sites. This was in accordance with the high adaptation capacity of the species in African urban environment [11, 35]. The two identified species, namely *An. coluzzii* and *An. gambiae s.s.*, were found in Cotonou and Natitingou, respectively. These data are consistent with previous studies conducted by Djegbè et al., Djogbenou et al. and Koukpo et al. [14, 36, 37].

Results showed a predominance of *An. coluzzii* species in the south of the country, while *An. gambiae s.s.* was the main species in the northern part. This species distribution can be explained by the varying climatic and environmental conditions as well as the ecological characteristics (physicochemical properties of larval habitats) provided by the cities [38]. *An. coluzzii* is known to be associated with urban environment and flooded sites, typified by extensive cultivation, as is the case in Cotonou, whereas *An. gambiae s.s.* was found in temporary, rain-dependent breeding sites, in Natitingou [18]. Regarding the physicochemical conditions, results showed that, salinity was the only physicochemical factors strongly associated with the presence of *An. coluzzii* in larval habitats found in Cotonou. These findings are consistent with previous phenotypic experiments that concluded that *An. coluzzii* had a higher tolerance to salinity than *An. gambiae s.s.* [39]. As one moves away from the coastal areas towards the inland regions, the salt content in larval habitats decreases, which could explain the progressive decrease in the proportions of *An. coluzzii* in the central and northern cities in favor of *An. gambiae s.s*. Furthermore, the positive association of *An. coluzzii* with turbidity, which is one of the physical determinants of water pollution, clearly demonstrated the adaptive capabilities of this species to more or less polluted environments in highly urbanized settings [40, 41]. On the contrary, turbidity was one of the factors negatively affecting the presence of *An. gambiae s.s.* in this study.

Moreover, the results indicate that the larvae of *An. gambiae s.s.* are associated with higher total coliform loads in larval habitats compared to those of *An. coluzzii*. In contrast, the larval habitats of *An. coluzzii* present a higher load of fecal coliforms, with a significant impact on the presence of *An. coluzzii*. These results suggest that the presence of specific coliforms could influence the distribution of Anopheles mosquito species within larval habitats. The higher total coliform load observed in habitats hosting *An. gambiae s.s.* could indicate environmental conditions favorable to the development of this species, although this association is not statistically significant. On the other hand, the significant impact of fecal coliforms on the presence of *An. coluzzii* suggests a preference for habitats with higher levels of fecal contamination.

The assessment of adult Anopheles resistance to insecticides revealed a very high level of resistance to deltamethrin across the different study sites. This resistance to pyrethroids confirms the findings of other studies conducted in Benin [16, 19, 42, 43]. The high level of resistance of these malaria vectors to pyrethroids observed in Benin could be associated with environmental factors, such as pollution in these urban areas, which increase the level of xenobiotics in larval habitats. Additionally, external factors such as the extensive use of pyrethroid-based control measures in vector management, along with failure of farmers to adopt optimal agricultural pesticide management practices, continuously exert selective pressure on anopheline mosquitoes [44–46].

This resistance of *An. gambiae s.l* populations to pyrethroids, observed in others countries in West Africa [47, 48], is correlated with the high frequency of the *Kdr L1014F* mutation observed in the mosquitoes analyzed in the two cities. As in many other studies, this suggests that the *Kdr* mutation plays an important role in insecticide resistance of the pyrethroid family [38]. Furthermore, both species found in this study, namely *An. coluzzii* and *An. gambiae s.s.*, both possess the *Kdr L1014F* mutation at similar frequencies in different cities. The same observations have been made in previous studies conducted in the country [14, 37]. However, other authors have shown that this resistance mechanism could be multigenic, and the *kdr 1014 F* allele alone may not fully explain the entire variance of the resistance phenotype, as observed in the present study [49]. It is possible that in addition to the *kdr L1014F* mutation, other mutations could play a role, allowing mosquitoes to survive after exposure to a discriminating concentration of pyrethroids.

Furthermore, the high frequencies of the *Kdr L1014F* mutation observed were significantly correlated with some physicochemical parameters, depending on the studied cities. These included salinity, pH, conductivity, and TDS (Total Dissolved Solids). Despite the current lack of studies examining the direct influence of physicochemical factors on the development of resistance or resistance profiles in Anopheles mosquitoes, research conducted by Ononamadu et al. [50]. in Kano Metropolis in Nigeria, revealed a significant correlation between the frequency of the *kdr* mutation and physicochemical properties such as TDS, phosphate, sulfate, potassium, manganese, and iron. Kabula et al. [40]. demonstrated that mineral content and silica were the best discriminating parameters for the presence of the *Kdr* mutation in larval habitats of *An. gambiae s.s*. These findings suggest that anopheles mosquitoes tolerance to such physicochemical parameters could be associated with the resistance profile observed. However, the extent to which these water parameters are involved in the occurrence of the *kdr* mutation in *An. gambiae* is not well understood and need further investigations.

In addition to pyrethroid resistance, this study has revealed a low level of resistance in *An. gambiae* to carbamates (*Ace-1R*), confirming previous findings by Djènontin et al. [14]. Gnanguenon et al. [51]. , and Yadouleton et al. [43]. The allelic frequency of the Ace-1R resistance gene were 3% in Cotonou and 9% in Natitingou, which are higher than the frequencies reported in previous studies reporting a variation range from 1 to 2.5% [52, 53]. These results may indicate an increase in the frequency of the Ace-1R mutation in Benin [54]. The observed resistance to bendiocarb in the cities of Cotonou and Natitingou can also be attributed to various indoor residual spraying campaigns conducted in the Northwest and South of Benin between 2008 and 2018 as part of malaria vector control efforts, which might have facilitated the emergence of resistant vectors [55].

The findings of this study provide valuable insights into the complex interactions between Anopheles mosquitoes, their habitats, and insecticide resistance in urban sites. This information can be useful in targeted larval source management strategies aimed at reducing vector populations. Additionally, National Malaria Control Program (NMCP), using this data, can identify and prioritize breeding sites for interventions such as habitat modification or larvicide application. Moreover, this study highlights the need for malaria control program to take a proactive approach by considering rotation or combination of different classes of insecticides to effectively combat resistance.

## Conclusions

This study has established a relationship between specific biotic and abiotic factors of larval habitats and distribution of *An. gambiae s.s.* and *An. coluzzii*, as well as their resistance to insecticide in the cities of Cotonou and Natitingou. The results highlight the significant influence of ecological characteristics, leading to a preponderance of *An. coluzzii* in Cotonou and *An. gambiae s.s.* in Natitingou. Additionally, the study also revealed a high resistance to the tested insecticides and high frequency of *kdr* mutation in both Anopheles species. The association between the characteristics of larval habitats and the distribution of the *kdr* mutant allele suggested that molecular mechanisms, in combination with certain physicochemical factors, could explain the observed resistance to deltamethrin. These findings underscore the importance of an integrated approach involving environmental measures to combat malaria vectors in urban areas. By integrating these findings into its strategies, National Malaria Control Programme is expected to improve the effectiveness and sustainability of vector control efforts not only in Cotonou and Natitingou, but also in others cities with the same malaria challenges.

## Data Availability

The datasets generated and/or analysed during the current study are available here: 10.17605/OSF.IO/7CAE5.
